# Differential expression and correlation analysis of miRNA–mRNA profiles in swine testicular cells infected with porcine epidemic diarrhea virus

**DOI:** 10.1038/s41598-021-81189-5

**Published:** 2021-01-21

**Authors:** Xiaoqian Zhang, Chang Li, Bingzhou Zhang, Zhonghua Li, Wei Zeng, Rui Luo, Jiyue Cao, Guofu Cheng, Shengxian Fan, Qigai He

**Affiliations:** 1 State Key Laboratory of Agricultural Microbiology, Huazhong Agricultural University, Wuhan, 430070 China; 2 The Cooperative Innovation Center for Sustainable Pig Production, Huazhong Agricultural University, Wuhan, 430070 China; 3 College of Veterinary Medicine, Huazhong Agricultural University, Wuhan, 430070 Hubei Province China

**Keywords:** Cell biology, Mechanisms of disease, High-throughput screening, Cellular signalling networks, Computational biology and bioinformatics, Gene regulatory networks

## Abstract

The variant virulent porcine epidemic diarrhea virus (PEDV) strain (YN15) can cause severe porcine epidemic diarrhea (PED); however, the attenuated vaccine-like PEDV strain (YN144) can induce immunity in piglets. To investigate the differences in pathogenesis and epigenetic mechanisms between the two strains, differential expression and correlation analyses of the microRNA (miRNA) and mRNA in swine testicular (ST) cells infected with YN15, YN144, and mock were performed on three comparison groups (YN15 vs Control, YN144 vs Control, and YN15 vs YN144). The mRNA and miRNA expression profiles were obtained using next-generation sequencing (NGS), and the differentially expressed (DE) (*p-*value < 0.05) mRNA and miRNA were obtained using DESeq R package. mRNAs targeted by DE miRNAs were predicted using the miRanda algortithm. 8039, 8631 and 3310 DE mRNAs, and 36, 36, and 22 DE miRNAs were identified in the three comparison groups, respectively. 14,140, 15,367 and 3771 DE miRNA–mRNA (targeted by DE miRNAs) interaction pairs with negatively correlated expression patterns were identified, and interaction networks were constructed using *Cytoscape*. Six DE miRNAs and six DE mRNAs were randomly selected to verify the sequencing data by real-time relative quantitative reverse transcription polymerase chain reaction (qRT-PCR). Based on bioinformatics analysis, we discovered the differences were mostly involved in host immune responses and viral pathogenicity, including NF-κB signaling pathway and bacterial invasion of epithelial cells, etc. This is the first comprehensive comparison of DE miRNA–mRNA pairs in YN15 and YN144 infection in vitro, which could provide novel strategies for the prevention and control of PED.

## Introduction

As an acute enteric disease of piglets, porcine epidemic diarrhea (PED) is typically characterized by watery diarrhea accompanied by vomiting, dehydration and high mortality^1^. PED virus (PEDV), a member of the *Coronaviridae* family, is the etiological agent of PED and a single-stranded positive-sense RNA virus whose genome is approximately 28 kb in size. The genome includes at least seven open reading frames (ORFs) of which ORF1a and ORF1b encode nonstructural proteins. The other five ORFs encode four structural proteins and one accessory protein, namely spike (S) protein, ORF3, envelope (E) protein, membrane (M) protein, and nucleocapsid (N) protein in sequence^1^.

Since the late 2010s, a severe outbreak of PED has affected Asian, Europe and the Americas, and has caused great financial losses to the swine industry throughout the world^2–4^. The variant PEDV YN1 strain was isolated, passaged to 15 generations to obtain the variant virulent strain. This strain was passaged further for a total of 144 passages to obtain the attenuated, YN144^5^. In animal experiments, rather than inducing diarrhea in piglets, the YN144 strain induced innate immunity and neutralizing antibody responses, and provided protection to piglets challenged with a variant virulent strain^5^. In order to better understand the pathogenic mechanism of the variant PEDVs, a proteomics study was performed in vivo following the variant and attenuated strain infection, whose differentially expressed proteins are associated with immune function and stress responses^6^.

Accumulating studies have demonstrated that microRNA (miRNA) can regulate the signaling pathways of host cells to defend against viral infections^7^. miRNA is a single-stranded, noncoding RNA of approximately 18–24 nucleotide length and is derived from longer preprimary transcripts^8^. The target recognition sequence of a mature miRNA, called the seed region, is highly conserved and is located within positions 2–8 of the miRNA. miRNA can regulate the expression of target mRNAs by binding to the 3′ or 5′ untranslated region of target mRNA using its seed region and ultimately inducing mRNA degradation, thereby playing a crucial role in controlling post-transcriptional gene expression in almost all known physiological and pathophysiological processes^9^. miRNA has two distinct anti-viral mechanisms. First, miRNA directly targets viral mRNA^10^. Second, miRNA can activate the host immune responses to control viral replication^11^. Next-generation sequencing (NGS) has become a powerful tool to analyze the expression profiles of miRNA and mRNA^12^.

In this study, the differential expression and correlation analysis of the differentially expressed (DE, *p*-value < 0.05) miRNA and mRNA in swine testicular (ST) cells infected with the variant virulent PEDV strain (YN15) and the attenuated vaccine-like PEDV strain (YN144) were investigated to understand the roles of the miRNA–mRNA network in PEDV pathogenesis using NGS technology. These findings could lead to novel ideas for the prevention and control of PED.

## Results

### Replication kinetics of PEDV variant strains YN15 and YN144 in ST cells in vitro

**Figure 1:**
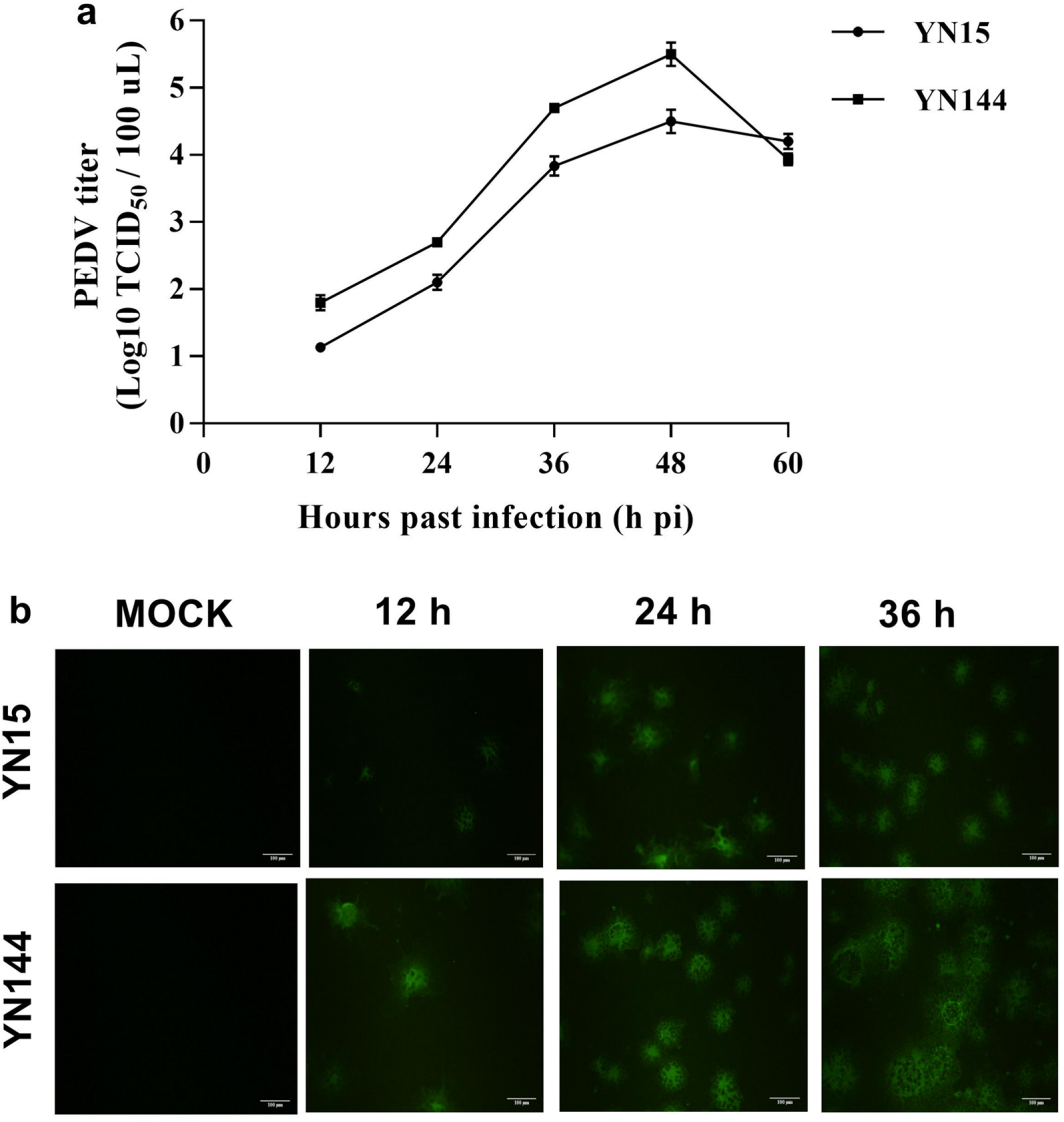
The growth curves of the two PEDV strains (YN15 and YN144) in ST cells (0.001 multiplicity of infection (MOI)). (**a**) The growth curves of the two PEDV strains (YN15 and YN144) in ST cells. (**b**) Confirmation of YN15 and YN144 replication in ST cells at 12, 24, and 36 h pi by IIFA. Mock-infected ST cells at 36 h pi were set as control. Scale bar = 100 μm.

Viral titers in ST cells were monitored at different time points after YN15 or YN144 infection (Fig. 1). The growth curves of the two PEDV strains, YN15 and YN144, in ST cells show that both strains peak at 48 h post-infection (pi), then gradually decline (Fig. 1a). In order to make the process of replication kinetics and identification of the intracellular PEDV (YN15 and YN144 strains) more visual, indirect immunofluorescence assay (IIFA) was used to track the intracellular PEDV strains. The intracellular PEDV replication over the first three 12 h interval time points was validated, and there was a high virus yield at 24 h pi (Fig. 1b). RNA samples were prepared using the cell pellets collected at 24 h pi for further small RNA and mRNA sequencing.

### Global mRNA expression patterns in ST cells after YN15 and YN144 infection

**Figure 2:**
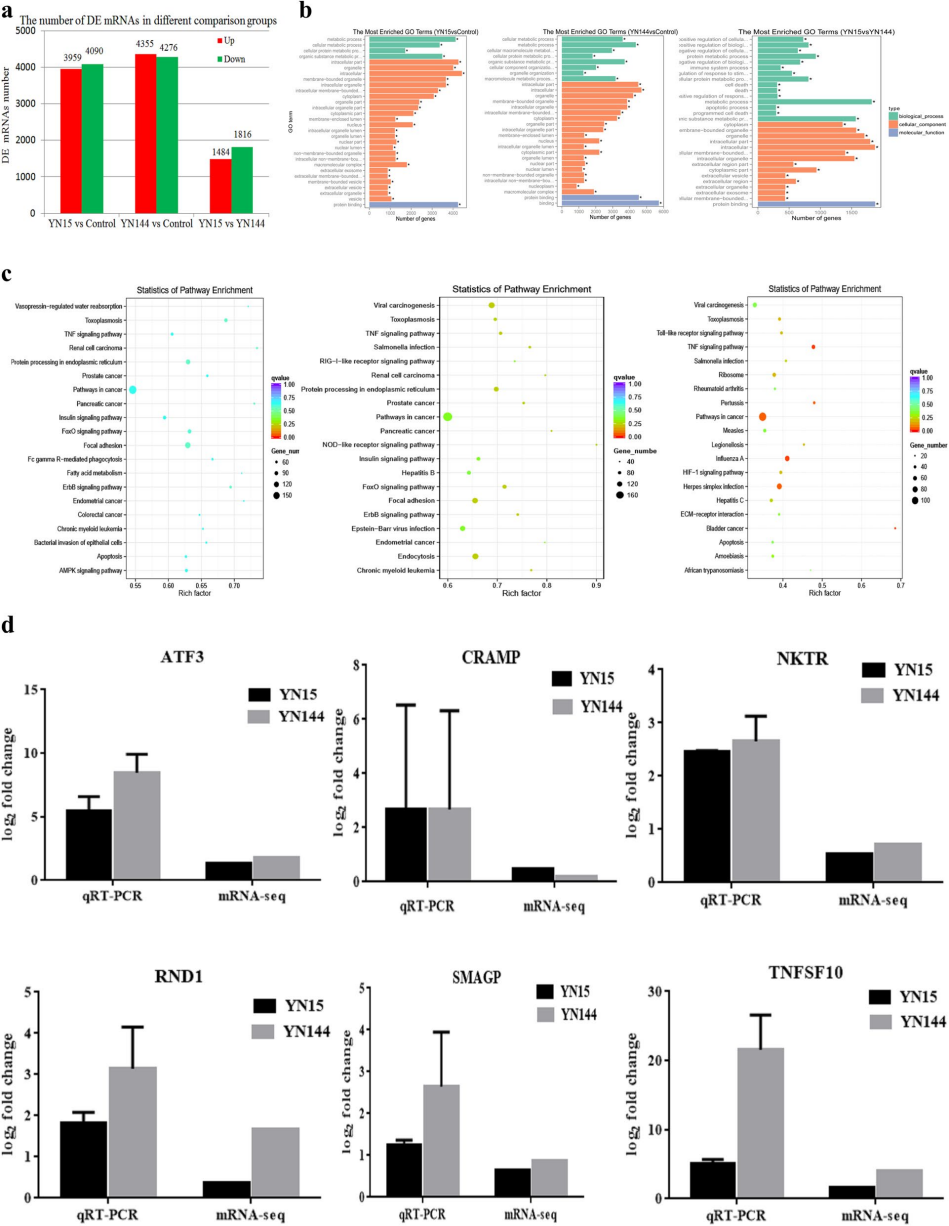
mRNAs expression profiles in PEDV-infected ST cells. (**a**) The bar graph shows the number of DE mRNAs in each one of the three comparison groups, red represents the amount of up-regulated DE mRNAs, green represents the count of down-regulated DE mRNAs (*p*-value < 0.05). (**b**) GO analysis of the DE mRNAs in PEDV infection ST. The three panels show the GO enrichment analysis of each of the top 30 significantly most DE mRNAs from the three comparison groups (YN15 vs Control, YN144 vs Control, and YN15 vs YN144), respectively. (**c**) KEGG analysis of the DE mRNAs in ST cells infected with PEDV. The three panels show the KEGG enrichment analysis of each of the top 20 significantly most DE mRNAs between YN15, YN144 and control groups, respectively. (**d**) Results of relative quantitative real-time reverse transcription polymerase chain reaction (qRT-PCR) analysis (alongside RNA-seq analysis) of *ATF3, CRAMP, NKTR, RND1, SMAGP and TNFSF10* in YN15- and YN144-infected ST cells, respectively. The relative expression of each mRNA was calculated using the 2^-ΔΔCt^ method. The data are presented as mean ± SEM from three independent experiments.

To investigate mRNA expression profiles during PEDV (YN15 or YN144) infection, gene transcripts were analyzed using RNA-seq, and the same total RNAs were used to construct nine sample sequence libraries. The quality assessment of the sequencing data is summarized in Supplementary Table S1. Among the clean reads, most were mapped to the *Sus scrofa* genome, and the sequence alignment results are shown in Supplementary Table S2. Genes were defined as DE when the *p*-value for the difference in expression was < 0.05 (*padj* < 0.05). Compared with the control group (uninfected ST cells), 8039 (3959 up-regulated and 4080 down-regulated) DE mRNAs were identified in the YN15 infection group and 8631 (4355 up-regulated and 4276 down-regulated) DE mRNAs were identified in the YN144 infection group. When the DE mRNAs in YN15 group were compared to those of YN144 group, 3310 (1494 up-regulated and 1816 down-regulated) DE mRNAs were commonly DE in both (Fig. 2a and Supplementary Table S3).

Gene Ontology (GO)-based analysis and Kyoto Encyclopedia of Genes and Genomes (KEGG) mapping were performed to explore the biological functions of all of the DE mRNAs. The most significantly enriched GO terms for target mRNAs in the three comparison groups are shown in Fig. 2b. All of the KEGG enrichment pathways are shown in Supplementary Table S4; among them each of the top 20 significantly enriched KEGG pathways is shown in Fig. 2c. Although the DE mRNAs differ depending on the specific experimental group comparison, most of them were involved in biological process (bp), cellular component (cc) and molecular function (mf). There were some commonly enriched GO terms, including protein binding, metabolic process, organic substance metabolic process, intracellular part, and organelle. In addition to the commonly enriched GO terms, the uniquely DE mRNAs in the YN15 vs Control comparison group, are involved in extracellular exosome, membrane-bounded vesicle, extracellular organelle, and vesicle. The uniquely DE mRNAs in the YN144 vs Control comparison group are involved in macromolecule metabolic process, cellular component organization, nucleoplasm, and binding. In the YN15 vs YN144 comparison group, the top 30 enriched GO terms for the DE mRNAs are involved in immune system process, regulation of cellular process, regulation of biological process, programmed cell death, apoptotic processes, and inflammatory response. According to the KEGG mapping of the DE mRNAs, most of the top 20 significant signaling pathways were different across the three comparison groups, except for pathway in cancer, tumor necrosis factor (TNF) signaling pathway and toxoplasmosis. The DE mRNAs are mostly involved in Fc gamma R-mediated phagocytosis, fatty acid metabolism, apoptosis, AMPK signaling pathway, and bacterial invasion of epithelial cells in the YN15 vs Control comparison group. While, the DE mRNAs in the YN144 vs Control comparison group are involved in viral carcinogenesis, RIG-I-like receptor signaling pathway, NOD-like receptor signaling pathway, chronic myeloid leukemia, and endocytosis. In the YN15 vs YN144 comparison group, the DE mRNAs are mostly involved in Toll-like signaling pathway, apoptosis, extracellular matrix protein (*ECM*)-receptor interaction, and the nuclear factor kappa-B (NF-κB) signaling pathway.

To confirm the results obtained from RNA-seq, six randomly selected mRNAs (activating transcription factor 3 *(ATF3),* cramped chromatin regulator homolog *(CRAMP),* natural killer cell triggering receptor *(NKTR),* Rho family GTPase 1 *(RND1),* small cell adhesion glycoprotein *(SMAGP) and* tumor necrosis factor superfamily member 10 *(TNFSF10)*) were measured using real-time relative quantitative reverse transcription polymerase chain reaction (qRT-PCR) and their changes in expression showed trends consistent with the RNA-seq results (Fig. 2d), indicating that the RNA-seq data are reflective of actual changes at the genetic level.

### Global miRNA expression patterns in ST cells after YN15 and YN144 infection

**Figure 3:**
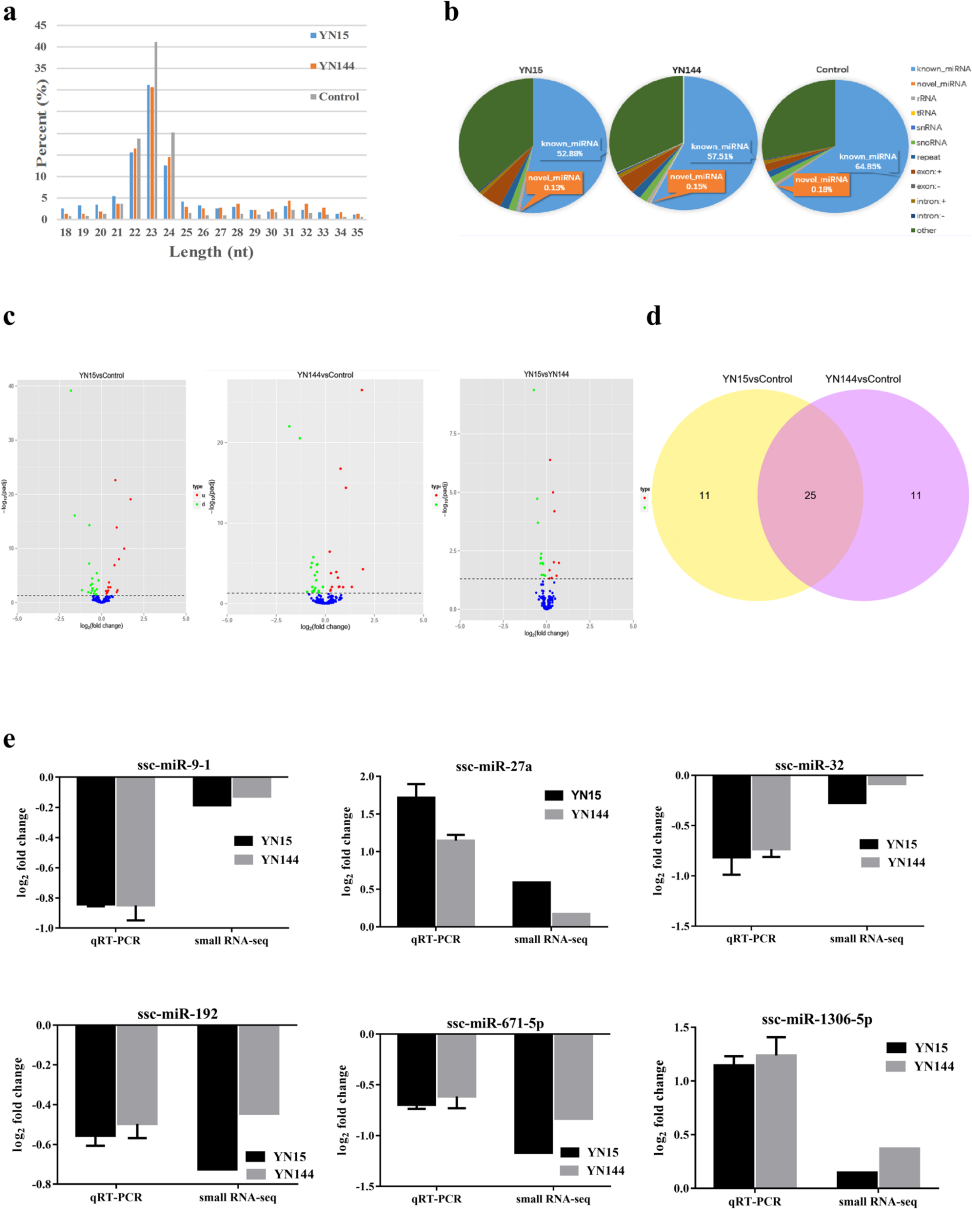
miRNA expression profiles in PEDV-infected ST cells. (**a**) Length of distribution of small RNA-seq reads. (**b**) Pie charts of small RNA-seq results showing the percentage of small RNA components in PEDV YN15-, YN144-, and mock-infected (Control) ST cells. (**c**) Volcano plots of up-regulated and down-regulated differentially expressed microRNAs (DE miRNAs) in three comparison groups (YN15 vs Control, YN144 vs Control, and YN15 vs YN144) (*p*-value < 0.05). (**d**) Venn diagram of the number of the commonly and uniquely DE miRNAs between the two comparison groups (YN15 vs Control and YN144 vs Control). Each number in a circle represents the number of the DE miRNAs found in each comparison group. The number in the overlapping area represents miRNAs commonly DE between the two comparison groups. The number in each non-overlapping area indicates the unique DE miRNAs in each comparison group. (*p*-value < 0.05). (**e)** Results of real-time relative qRT-PCR analysis (alongside RNA-seq analysis) of ssc-miR-9–1, ssc-miR-27a, ssc-miR-32, ssc-miR-192, ssc-miR-671-5p and ssc-miR-1306-5p fold-change in the two comparison groups (YN15 vs Control and YN144 vs Control). The relative expression of each miRNA was calculated using the 2^-ΔΔCt^ method. The data are presented as mean ± SEM from three independent experiments.

Raw reads from the libraries derived from YN15, YN144 and control groups were generated. Clean reads were obtained after filtration. The clean reads successfully mapped to the reference genome sequence of *Sus scrofa*, as shown in Supplementary Table S5. The length of the majority of clean reads obtained were 22–24 nucleotides (nt), and the size of 23 nt was the most common (Fig. 3a). The percentage of nucleic acid represented by miRNAs in YN15, YN144, and control groups is shown in Fig. 3b. The numbers of mapped known and novel miRNAs are listed in Supplementary Table S6. After BLASTN analysis by *miRBase 20.0* with *Sus scrofa* miRNAs, 275 known and 279 novel mature miRNAs were obtained (Supplementary Table S7).

Identification of DE miRNAs was performed based on *p*-value < 0.05. Based on the volcano plots, three DE miRNA clusters emerged (Fig. 3c). Among these DE miRNAs, YN15 infection was found to increase the expression of 15 miRNAs and decrease the expression of 21 miRNAs in ST cells, YN144 infection up-regulated 15 miRNAs and down-regulated 21 miRNAs, and 22 miRNAs, 9 up- and 13 down-regulated, were identified when comparing ST cells infected with YN15 and ST cells infected with YN144 (Fig. 3c and Supplementary Table S8). Among all of DE miRNAs, seven novel miRNAs were discovered (Supplementary Table S8).As shown in the Venn diagram (Fig. 3d), 25 commonly and 22 uniquely DE miRNAs were identified between the YN15 vs Control comparison group and the YN144 vs Control comparison group. All 25 commonly DE miRNAs exhibited the same trend in expression, i.e., 11 up-regulated and 14 down-regulated miRNAs (Supplementary Table S9).

To identify the data obtained from small RNA-seq, six randomly selected miRNAs (ssc-miR-9–1, ssc-miR-27a, ssc-miR-32, ssc-miR-192, ssc-miR-671-5p and ssc-miR-1306-5p) were evaluated by real-time relative qRT-PCR (Fig. 3e). The comparable results indicate that the data obtained by RNA-seq were valid.

### Prediction and functional characterization of target mRNAs of DE miRNAs

**Figure 4:**
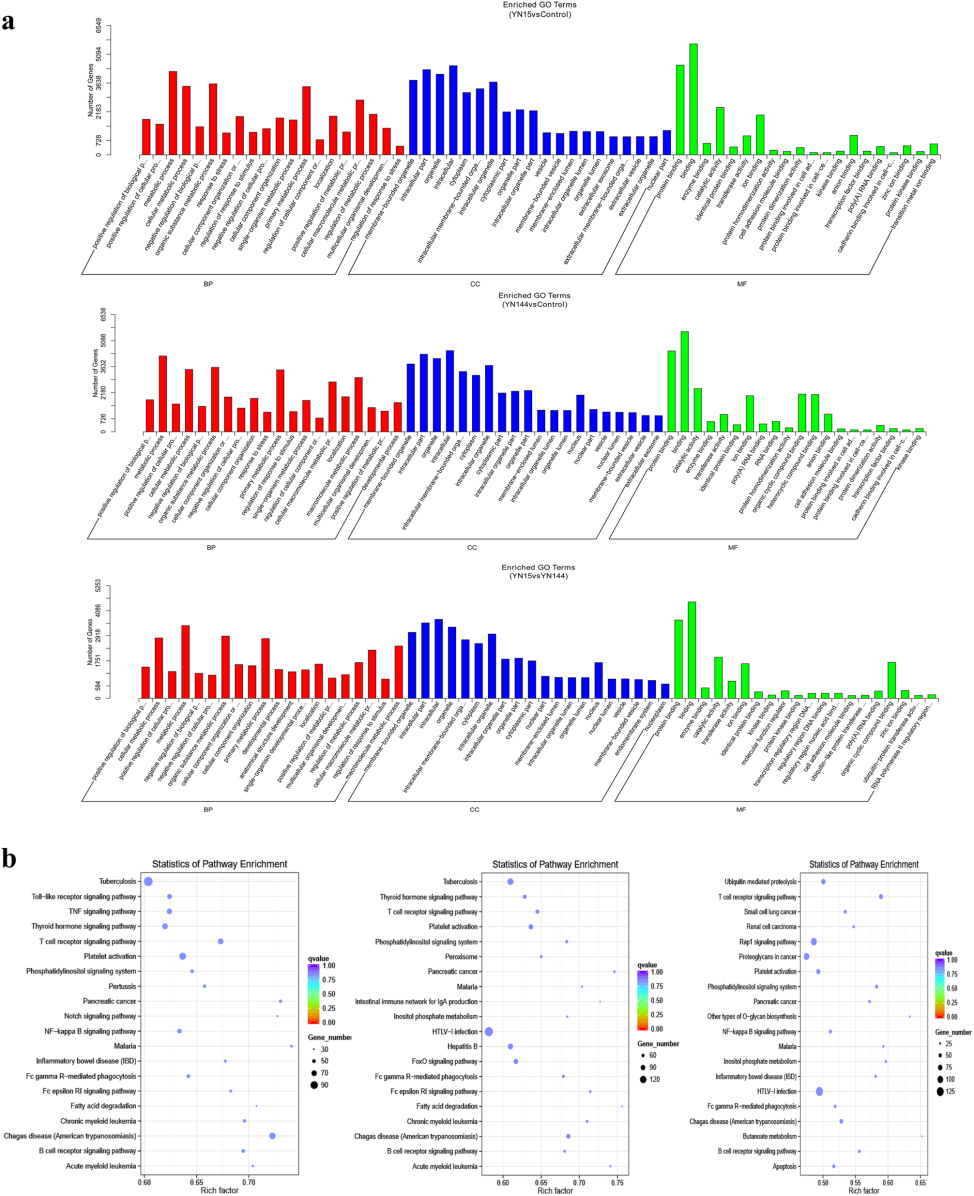
DE miRNAs and their predicted target mRNAs in PEDV-infected ST cells. (**a**) GO analysis of the predicted target genes of the DE miRNAs from the three comparison groups. (**b**) KEGG analysis of the predicted target genes of the DE miRNAs from the three comparison groups. Each panels indicates the top 20 significantly enriched pathways identifed by KEGG analysis of each of the three comparison groups (Left, YN15 vs Control; Middle, YN144 vs Control; Right, YN15 vs YN144).

The target mRNAs of DE miRNA identified in the three comparison groups (YN15 vs Control, YN144 vs Control, and YN15 vs YN144) were predicted using the miRanda algorithm (Table. 1). GO and KEGG analyses were performed to characterize the functions of the predicted target mRNAs. The most significantly enriched GO terms of target mRNAs between the three comparison groups are involved in the biological processes of bp, cc and mf (Fig. 4a). All of the enriched pathways identified through KEGG mapping are shown in Supplementary Table S10; among them, the top 20 significantly enriched pathways represented by predicted target mRNAs from each comparison group were identified (Fig. 4b). In the YN15 vs Control comparison group, the target mRNAs were associated with immune- and bowel disease-related signaling pathways, including T cell receptor signaling pathway, Toll-like receptor signaling pathway, and inflammatory bowel disease (IBD), etc. In the YN144 vs Control comparison group, the enriched genes were involved in immune-related signaling pathways, including T cell receptor signaling pathway, intestinal immune network for IgA production, human T-cell leukemia virus type 1 (HTLV-1) infection, Fc gamma R-mediated phagocytosis, and B cell receptor signaling pathway. When YN15 and YN144 infected cells were compared, the target mRNAs of DE miRNAs were almost all connected to immune- and bowel disease-related signaling pathways, either IBD, Rap1 signaling pathway, HTLV-1 infection, Fc gamma R-mediated phagocytosis, T cell receptor signaling pathway, or the NF-κB signaling pathway.

**Table 1:** Number of DE miRNAs and their target mRNAs.

Comparison group name	Up-regulated miRNAs	Predicted target mRNAs	Down-regulated miRNAs	Predicted target mRNAs
YN15 vs Control	15	26,168	21	38,908
YN144 vs Control	15	29,614	21	36,906
YN15 vs YN144	9	16,042	13	20,102

Table shows the numbers of up-regulated miRNA and down-regulated miRNA, and their target mRNAs in each of the three comparison groups, respectively.

### Joint functional analysis of DE miRNAs and DE target mRNAs

**Figure 5:**
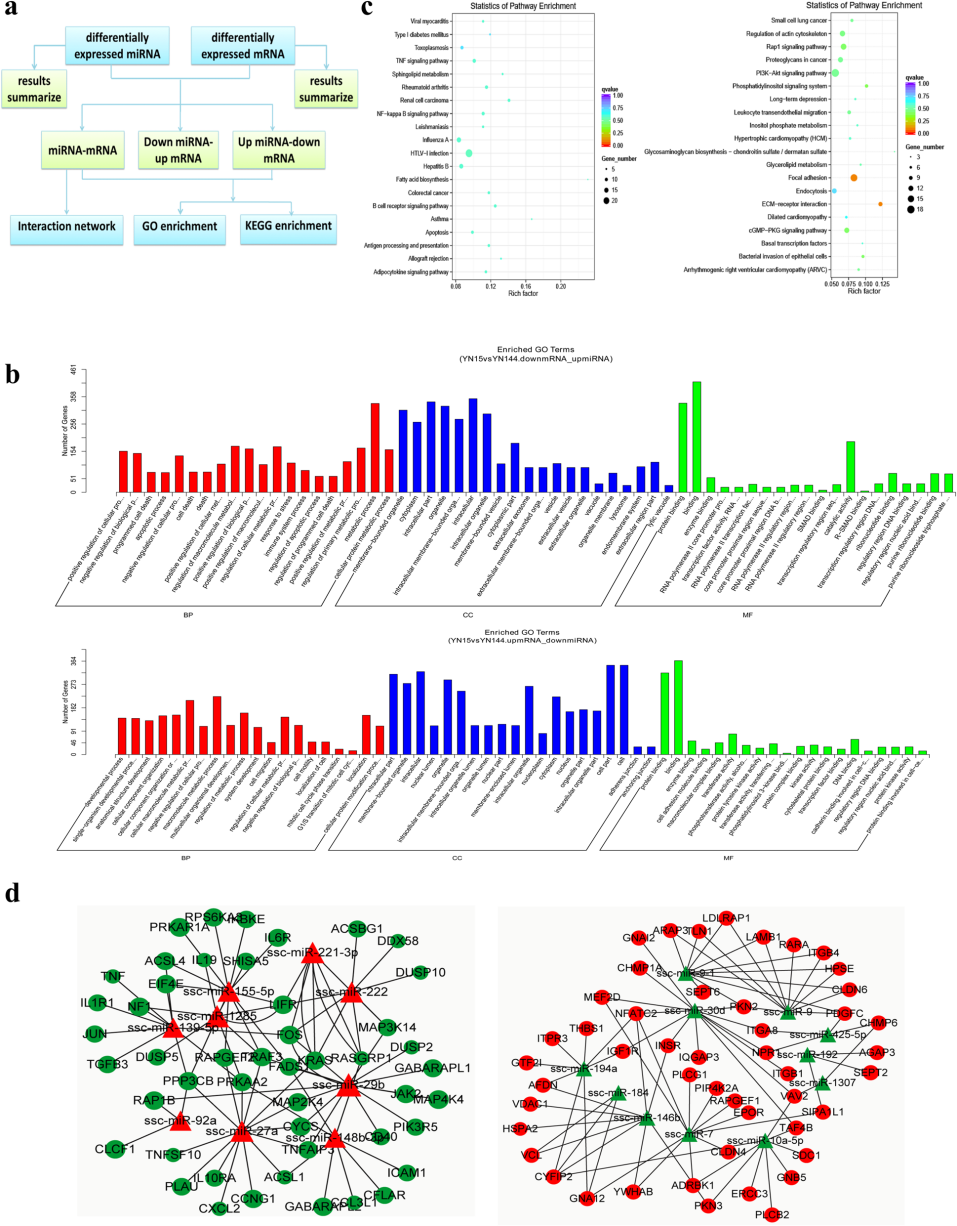
Joint functional analysis of DE miRNA and inversely DE target mRNA in PEDV-infected ST cells. (**a**) Flow diagram of joint analysis of DE miRNA–mRNA pairs in the three comparison groups. (**b**) GO analysis of DE miRNA–mRNA pairs between YN15 infection and YN144 infection. The top panel shows the GO enrichment analysis of up-regulated miRNA-down-regulated mRNA pairs, and the lower panel shows the GO enrichment analysis of down-regulated miRNA-up-regulated mRNA pairs during YN15 infection compared with YN14 infection. (**c**) KEGG analysis of the genes of the DE miRNA–mRNA pairs between YN15 infection and YN144 infection. The left panel shows the KEGG pathways of the top 20 significantly up-regulated miRNA-down-regulated mRNA pairs, and the right panel shows the KEGG pathways of the top 20 significantly down-regulated miRNA-up-regulated mRNA pairs during YN15 infection compared with YN144 infection. (**d**) The DE miRNA–mRNA interaction networks in PEDV-infected ST cells. The left panel shows the significant interactions of the up-regulated miRNA-down-regulated mRNA pairs, and the right panel shows the significant interactions of the down-regulated miRNA-up-regulated mRNA pairs during YN144 infection in comparison with YN15 infection. *p*-value < 0.05.

Using the strategy illustrated in Fig. 5a, a large number of DE miRNA–mRNA pairs were identified (*p*-value < 0.05). Our work indicated that one mRNA can be targeted and regulated by multiple miRNAs, and one miRNA can regulate a large number of mRNAs, simultaneously. With the exception of one miRNA that had a negative correlation with its target mRNAs, the miRNA and target mRNA expression profiles from ST cells infected with YN15 or YN144 were positively correlated. Our research also revealed DE miRNAs with target mRNAs that change in the opposite direction and form inversely correlated DE miRNA–mRNA interaction pairs, including up-regulated miRNA-down-regulated mRNA pairs and down-regulated miRNA-up-regulated mRNA pairs (Supplementary Table S11). The numbers of inversely regulated DE miRNA–mRNA pairs in PEDV-infected ST cells in the three comparison groups are shown in Table 2.

**Table 2:** Number of inversely DE miRNA–mRNA pairs.

Comparison group name	Up-regulated miRNA-down-regulated mRNA pairs	Down-regulated miRNA-up-regulated mRNA pairs	Total DE miRNA–mRNA pairs
YN15 vs Control	5739	8401	14,140
YN144 vs Control	6847	8520	15,367
YN15 vs YN144	1693	2078	3771

Table displaying the numbers of DE miRNA–mRNA pairs between the three comparison groups.

A joint analysis of the interactions between DE miRNA–mRNA pairs with a negative correlation was performed. When comparing ST cells infected with YN15 and cells infected with YN144, GO and KEGG enrichment among up-regulated and down-regulated DE target mRNAs were performed. The top 20 most significantly enriched GO terms are involved in the biological functions of bp, cc and mf (Fig. 5c). All KEGG enrichment pathways of the DE target mRNAs are shown in Supplementary Table S12. The 20 most significantly enriched KEGG mapped pathways of the inversely regulated DE miRNA–mRNA pairs are shown in Fig. 5d. These DE miRNA–mRNA pairs were jointly analyzed according to functionally enriched GO terms and enriched KEGG pathways, and the two interaction networks were formed using *Cytoscape* software (Fig. 5e). One interaction network shows that the differentially up-regulated miRNA-down-regulated mRNA pairs during YN15 infection in comparison with YN144 infection are involved in focal adhesion, endocytosis, regulation of action cytoskeleton, PI3K-Akt signaling pathway, and bacteria invasion of epithelial cells. The second interaction network shows that the differentially down-regulated miRNA-up-regulated mRNA pairs are involved in the NF-κB signaling pathway, TNF signaling pathway, B cell receptor signaling pathway, Toll-like receptor signaling pathway, T cell receptor signaling pathway, Janus kinase/signal transduction and activator of transcription (JAK-STAT) signaling pathway, and apoptosis.

## Discussion

miRNA–mRNA interaction networks have emerged as crucial regulators in virus-host interactions, and NGS technology has become a powerful tool to analyze the expression profiles of miRNA and mRNA^12^. In view of the significant differences in pathogenesis and epigenetic mechanisms between the variant virulent PEDV strain YN15 and the attenuated vaccine-like PEDV strain YN144 in vivo, we suppose that miRNA and mRNA may play essential roles in PEDV virulence. This is the first reported comparative analysis of ST cell responses to infection with a virulent and attenuated strain of PEDV using small RNA-seq and mRNA-seq to explore the molecular mechanisms in the virus-host interaction.

According to our sequencing data, the ST cell transcriptional responses to infection with PEDV YN15 (virulent) and YN144 (attenuated) strains were very different in the early stage of infection (24 h pi, MOI = 0.001). According to the comprehensive GO and KEGG pathway enrichment analysis of DE miRNA–mRNA pairs with negatively correlated expression patterns in the YN15 vs YN144 comparison group, the main signaling pathways enriched were involved in host immunity and viral pathogenicity.

The main innate immune-related signaling pathways including NF-κB signaling pathway and JAK-STAT signaling pathway excited our interest. The NF-κB signaling pathway plays a crucial part in host immunity during viral infections^13^. The PEDV N protein is capable of inhibiting the activation of this signaling pathway activation in HEK-293 T cells^14^. Our sequencing data shows that *NFκB1*, a member of the NF-κB family of transcription factors that can activate the NF-κB signaling pathway, was substantially up-regulated in ST cells during YN144 infection (YN144 vs Control, *p*-value = 3.603E−31), while its expression during YN15 infection was moderate (YN15 vs Control, *p*-value = 5.82E−9).

The JAK-STAT signaling pathway also plays a major role in host innate immunity, especially as it pertains to inflammatory pathogenesis as seen in IBD^15^. In this work, cytokines related to this signaling pathway were found to be up-regulated during variant PEDV strain infection, especially during YN144 infection. The phosphorylation and translocation of *STAT1*, *STAT2* and *STAT3* promotes the antiviral ability of cells by up-regulating the transcription of interferon (*IFN*) -stimulated genes (*ISG*) to fight viral infection^16^. Our deep sequencing data showed that *STAT1* and *STAT3* were markedly up-regulated in YN144-infected ST cells (YN144 vs Control, *STAT1*: *p-*value = 9.0857E−17, and *STAT3*: *p-*value = 3.00E−11) compared with YN15-infected ST cells (YN15 vs Control, *STAT1*: *p-*value = 6.43E−12, and *STAT3*: *p-*value > 0.05). *STAT2* expression displayed no obvious changes during YN144 infection (YN144 vs Control, *p-*value > 0.05), but was notably decreased during YN15 infection (YN15 vs Control, *p-*value = 6.81E−08). As key members of this signaling pathway, both the oligoadenylate synthetase (*OAS*) protein family (*OAS1*, *OAS2*, and *OASL*) and the Mx dynamin-like GTPases (*MX1* and *MX2*) induced by type I and type III *IFN* participate in antiviral activity and inhibit virus replication^17,18^. Our research shows that the expressions of the above genes can be substantially activated at early stages of YN144 infection, while their expressions were moderate at early stages of YN15 infection (YN15 vs YN144, *OAS1*: *p*-value = 1.6782E−130, *OAS2*: *p*-value = 1.8062E−247, *OASL*: *p*-value = 3.51E−11, *MX1*: *p*-value = 3.94E−25, and *MX2*: *p*-value = 8.38E−252).

In this work, the NF-κB signaling pathway was found to be regulated by ssc-miR-155-5p, ssc-miR-29b, and ssc-miR-139-5p, and the JAK-STAT signaling pathway was regulated by ssc-miR-155-5p, ssc-miR-29b, ssc-miR-1307, ssc-miR-10a-5p, and ssc-miR-30d. All of miR-155-5p, miR-139-5p and miR-29b have been proved to have immense effects on inflammation and negatively regulate the innate immune responses to various microorganisms^19–21^. For example, miR-155 and miR-139-5p can constitute a negative feedback loop in the NF-κB signaling pathway by targeting multiple key proteins, leading to the repression of NF-κB activation in response to viral or microbial stimuli^19,20^. The expression of miR-155 was enhanced in microglial cells during Japanese encephalitis virus (JEV) infection, and JEV-induced *IFN-β* as well as downstream ISG mRNA expression was significantly reduced in microglial overexpressing miR-155^19^.The knock-out or inhibition of miR-155 decreases hepatitis C virus (HCV) viremia in children with leukemia^22^. miR-29b is capable of inhibiting the JAK-STAT signaling pathway by *IFN-γ* in CD8 + T cells and TH1 effector cells^21^. Interestingly, our sequencing data showed that ssc-miR-155-5p and ssc-miR-139-5p were markedly up-regulated in YN15-infected ST cells compared with YN144-infected ST cells (YN15 vs YN144, ssc-miR-155-5p: *p*-value = 4.991E-03, and ssc-miR-139-5p: *p*-value = 1.671E-3). Ssc-miR-29b was significantly up-regulated in ST cells infected with YN15, but down-regulated in cells infected with YN144. These differences could explain our previous results that YN144 can more effectively activate the host immune response than YN15^5^.

A particularly noteworthy phenomenon was observed for the expression patterns of ssc-miR-1307, ssc-miR-10a-5p and ssc-miR-30d. All three were markedly down-regulated in ST cells during YN15 infection, but were significantly up-regulated during YN144 infection (shown in Supplementary Table S8). Interestingly, many studies have shown that all three play crucial roles in promoting host innate immunity during viral infection^23–25^. Qi et al. have provided evidence that miR-1307 can be significantly up-regulated in PK-15 cells after infection with foot-and-mouth disease virus^23^. The overexpression of this miRNA greatly up-regulates the expression of *IFN-β*, *ISG54*, *2′,5′-OAS*, and *NF-κB* subunit *p65/RELA*, and activates the innate immune response, including the NF-κB and JAK-STAT signaling pathways, at early stages of viral infection^23^. Other research has demonstrated that miR-10a is highly expressed in the intestines and plays a crucial role in the host immune response to the microbiota in a mouse model, and the intestinal microbiota negatively regulates miR-10a expression by targeting *IL-12/IL-23p40*^24^. The miR-30 family plays an important role in the immune response during viral infection^28^. miR-30b and miR-30c are markedly up-regulated with *IFN-α* treatment and inhibit HCV replication in Huh 7.5 cells^25^.

Interestingly, we found that the above DE miRNAs are all involved in host immune action, although through different means and pathways. All of the DE miRNAs that were up-regulated during YN144 infection and down-regulated during YN15 infection are capable of activating the host immune response. Even more surprising, the same phenomenon occurs among all commonly up-regulated miRNAs that the expression levels of the up-regulated miRNAs during YN15 infection were significantly higher than during YN144 infection, and all of them have been reported to inhibit the NF-κB or JAK-STAT signaling pathway. Therefore, we predict that the above DE miRNAs may play an indispensable role in regulating the host innate immunity during early PEDV infection in vitro, and the above differences may be some of the reasons that YN15 and YN144 can cause different immunity action in vitro and vivo.

In contrast, YN15 can invade intestinal epithelial cells and cause serious intestinal injury and inflammation, leading to serious diarrhea in piglets. It is worth noting that diarrhea in piglet is often caused by viral-bacterial co-infections in the farm setting^26^. Coincidentally, the GO and KEGG enrichment analysis of up-regulated mRNA-down-regulated miRNA pairs in YN15-infected ST cells compared with YN144-infected ST cells, showed that the major signaling pathways represented include focal adhesion, regulation of action cytoskeleton, endocytosis, PI3K-Akt signaling pathway, and bacterial invasion of epithelial cells. All of these pathways are closely related to pathogenicity, and in particular, the destruction of host cell structure during microbial invasion^27^.

Integrin (*ITG*), vinculin (*VCL*), and fibronectin (*FN1*) are associated with these signaling pathways. *ITGs* are heterodimeric proteins expressed on the cell surface that act as receptors for the interaction between cells and microorganisms and are exploited by microorganisms to gain entry into host cells^28^. *ITGαvβ3* is an entry receptor for PEDV infection in *Vero E6* and porcine intestinal epithelial cells^29^. During enterohemorrhagic *E. coli* (*EHEC*) *O157:H7* infection, *ITGβ1* on the surface of the intestinal epithelia acts as receptors for intimin expressed by EHEC, to promote bacterial infection^30^.

Both *talin* and *VCL* are actin-cytoskeleton-associated, adapter proteins for the dynamic interaction between *ITG* and actin cytoskeleton to generate focal adhesions between cells and *ECM* and establish bacterial and viral infections^31^. For example, Ebola virus infection promotes *talin-VCL* expression^31^. *IpaA* secreted by *Shigella* interacts with *VCL* to initiate the formation of focal adhesion-like structures required for the efficient bacterial invasion of epithelial cells^32^. *FN1* can polymerize to form a linear and branched meshwork on the cell surface, and a vast number of bacteria and viruses, such as *Staphylococcus aureus* and Influenza A Virus, express *FN*-binding proteins to promote cellular invasion^33–35^.

The PI3K-Akt signaling pathway is activated by variety of pathogens leading to accentuate diseases^36^. For example, activation of this signaling pathway promotes actin rearrangement, leading to the host inflammatory response that persists in IBD^37^. Additionally, cell destruction caused by viruses can expose cryptic receptors to promote bacteria adhesion and it is well known, that some symbiotic gut microbiota and invasive intestinal pathogens invade epithelial cells, and cause extensive intestinal damage^38–40^. Transmissible gastroenteritis virus (TGEV) infection can expose the adhesion proteins of intestinal epithelial cells, thereby providing the attachment sites for enterotoxigenic *E. coli* K88 (*ETEC*) invasion, inducing an epithelial–mesenchymal transition-like phenotype, and resulting in virus-bacteria co-infection^41^. Like TGEV, PEDV disrupts porcine intestinal epithelial cells; however, the mechanism of PEDV and bacteria co-infection is not clear^40^. Interestingly, paramyxovirus and *Streptococcus pneumonia* co-infection can only be supported by the highly pathogenic virus strains and not by the low pathogenic virus strains^42^. In addition, *mTOR*, a downstream factor of the PI3K-Akt signaling pathway, can inhibit T helper cell differentiation and thus suppress the host immune response^43^.

Importantly, the above phenomenon is reflected in our sequencing data demonstrationg that YN15 causes significantly greater activation of the above signaling pathways than dose YN144, as measured by the up-regulation of effector genes encoding *mTOR*, *FN1*, *VCL*, *ITGα3*, *ITGα5*, *ITGα6*, *ITGα8*, *ITGβ1*, and *ITGβ4* (YN15 vs YN144, *p*-value = 7.98E-06, *p*-value = 3.27E-04, *p*-value = 8.96E-08, *p*-value = 5.25E-12, *p*-value = 3.27E-04, *p*-value = 9.61E-20, *p*-value = 2.04E-12, *p*-value = 1.41E-03, and *p*-value = 2.52E-12, respectively) in ST cells. This may provide an explanation as to why YN15 causes serious intestinal inflammation and pathological damage, whereas YN144 does not.

We found that several DE miRNAs regulate these pathogenicity-related signaling pathways, including ssc-miR-9, ssc-miR-30d, ssc-miR-425-5p, and ssc-miR-184. miR-9-3p has been shown to suppress cell proliferation, migration and invasion via the down-regulation of *FN1*, *ITGβ1*, *ITGα5*^44^. In the present study, YN15 exhibited greater inhibition of ssc-miR-9 expression and greater activation of *FN1*, *ITGβ1*, and *ITGα5* expression than the YN144 strain. These results may provide us with new insights into how to inhibit PEDV replication and secondary bacterial infection. To promote cell invasion and metastasis, miR-425-5p could activate *ITGβ1*expression through the suppression of suppressor of cancer cell invasion (*SCAI*) expression^45^. It is worth mentioning that miR-184 is normally found in abundance in healthy individuals; however, down-regulation of miR-184 leads to epithelial thinning and prolonged wound healing, possibly contributing to pathology^46^. Our sequencing data show that ST cells infected with YN15 produce a more pronounced decrease in the expression of these miRNAs than when infected with YN144 (ssc-miR-9: *p*-value = 3.624E-03, ssc-miR-425-5p: *p*-value = 7.41E-04, and ssc-miR-184: *p*-value = 3.92E-07). Overexpression of miR-30 in breast cancer cells inhibits the expression of a number of genes associated with invasiveness (e.g., *ITGα5, ITGβ3*) and tumor cell invasion in vitro^47^. Our sequencing data show that ssc-miR-30d were up-regulated during YN144 infection and down-regulated during YN15 infection.

In summary, PEDV infection can activate and inhibit various of genes in the same signaling pathway, and the same gene can simultaneously play different roles in different signaling pathways and present different expression ability in different stage. Based on our sequencing data, genes enrichment analysis, and information from the literature, we speculate that YN15 infection would cause more serious pathological damage, and YN144 infection would markedly activated host immune responses in vivo and vitro, which correspond well with our previous studies in piglets and prediction that YN144 does not causes clinical symptoms, but still results in effective antibody production comparable to levels achieved by vaccination^5^. All differences are very attractive in helping researchers unravel a new antiviral mechanism of PEDV infection, and therefore provide researchers with further avenues to the porcine industry.

## Materials and methods

### Cells and viruses

ST cells were purchased from American Type Cultrue Collection (ATCC) and cultured in Dulbecco’s Modified Eagle’s Medium (DMEM, Invitrogen), supplemented with 8% Fetal Bovine Serum (BI) at 37 °C under 5% CO_2_. The variant virulent strain,YN1, was isolated from a sucking piglet with acute diarrhea, and was passaged to 15 generations in *Vero cells*, thus obtaining the virulent strain (Accession no. KT021228)^5^. The attenuated strain, YN144 (Accession no. KT021232), was obtained by passaging the same parent strain, YN1, to 144 generations in *Vero cells*. The detailed attenuation method and genomic comparison results were reported in our previous study^5^.

### Virus infection

ST cells were cultured for approximately 24 h to 90% confluence and were washed three times with serum-free DMEM. The titers of YN15 and YN144 in ST cells were 10^4.1^ median tissue culture infective dose (TCID_50_)/100 μL and 10^5.5^ TCID_50_/100 μL. Three experimental groups were designed; YN15-, YN144-, and mock-infected ST cells. Each group contained three independent biological replicates, which were named Control 1, Control 2, Control 3, YN15-1, YN15-2, YN15-3, YN144-1, YN144-2 and YN144-3. ST cells were infected separately with YN15 or YN144 at a multiplicity of infection (MOI) of 0.001 for 24 h. Mock-infected ST cells were used as control. After 1 h incubation at 37 °C under 5% CO_2_, unbound viruses were washed and discarded three times using serum-free DMEM. The cells were then incubated with serum-free DMEM containing 8 μg/mL trypsin (Invitrogen).

### Indirect immunofluorescence assay (IIFA)

ST cells infected with YN15 and YN144 PEDV strains were fixed with cold ethanol at 12, 24, and 36 h pi, respectively, and incubate with mouse monoclonal antibody against PEDV S protein for 1 h at 37 °C. Then, ST cells were washed three times with PBS before further incubation with Alexa Fluor 488-conjugated donkey anti-mouse IgG (H + L) (Thermo Fisher) in the dark. After washing five times with PBS, the images were captured under a fluorescence microscopy. Mock-infected ST cells at 36 h pi were set as control.

### RNA isolation and sample preparation

Total RNA was extracted from each biological repoicate of viral-infected or mock-infected ST cells using Trizol reagent (Invitrogen) and Polyacryl Carrier (MRC) at 24 h pi. RNA degradation and contamination were monitored on 1% agarose gals. RNA purity, integrity, and concentration were measured using the NanoPhotometer spectrophotometer (IMPLEN), the RNA Nano 6000 Assay Kit of the Aglient Bioanalyzer 2100 system (Aligent Technologies) and Qubit RNA Assay Kit in Qubit 2.0 Flurometer (Life Technologies). Only RNA samples with a ratio of absorbance (260/280 nm) > 1.8 and RNA integrity numbers (RINs) > 7 were used for RNA profiling.

### Library preparation and sequencing of mRNA

The library preparation and sequencing analyses of RNA were performed by Novogene Company (Bejing, China). A total quantity of 3 μg RNA per sample was used as input for the RNA sample preparations. Sequencing libraries were generated using NEBNext Ultra RNA Library Prep Kit for Illumina (NEB, USA) and index codes were added to attribute sequences to each sample. In order to select cDNA fragments of 150–200 bp in length, library fragments were purified using the AMPure XP system (Beckman Coulter, Beverly, USA). The PCR products were purified (AMPure XP system) and library quality was assessed on the Agilent Bioanalyzer 2100 system.

The clustering of the index-coded samples was performed on a cBot Cluster Generation System using TruSeq PE Cluster Kit v3-cBot-HS (Illumina). The library preparations were then sequenced on an Illumina HiSeq platform and 125 bp/150 bp paired-end reads were generated. The index of the reference genome was constructed using Bowtie v2.2.3 and paired-end clean reads were aligned to the reference genome using TopHat v2.0.12. HTSeq v0.6.1 was used to count the read numbers mapped to each gene. Then, fragments per kilobase of exon model per million reads mapped (FPKM) of each gene was calculated based on the length of the gene and numbers of reads mapped to the gene^48^.

### Library preparation, sequencing, prediction and analysis of miRNA

A total of 3 μg RNA per sample was used as input for each small RNA library. NEBNext Multiplex Small RNA Library Prep Set for Illumina (NEB, USA) was used to generate the sequencing libraries and index codes that were added to attribute sequences to each sample. Library quality was assessed on the Agilent Bioanalyzer 2100 system using DNA High Sensitivity Chips. After cluster generation of the index-coded samples, performed on a cBot Cluster Generation System using TruSeq SR Cluster Kit v3-cBot-HS (Illumina), the library was sequenced on an Illumina HiSeq 2500/2000 platform, and 50 bp single-end reads were generated.

The small RNA tags were mapped to the *Sus scrofa* sequence by Bowtie^49^. The miRBase20.0 database was used as the reference, and modified software mirdeep2 and sRNA-tools-cli were used to obtain potential miRNA and predict their secondary structures^50^. The available software miREvo and mirdeep2 were integrated to predict novel miRNAs^50,51^.

To explore the occurrence of the miRNA families identified in this study in other species, miFam.dat (http://mirbase.org/ftp.shtml) was used to search for known miRNA families, and novel miRNA precursors were submitted to Rfam (http://rfam.sanger.ac.uk/search/) to search for Rfam families.

miRNA expression levels were estimated by TPM (transcript per million) using the following criterion- normalization formula: normalized expression = mapped read count/ total reads × 1000000^52^.

### Differential expression of miRNA and mRNA, and target gene prediction

Analysis of DE miRNA from the three experimental groups (YN15, YN144 and control) was performed using the DESeq R package (1.8.3). Differentially expressed mRNAs from the three comparison groups were analyzed using the DESeq R package (1.18.0). The *p*-value was adjusted using the Benjamini & Hochberg method. A corrected *p*-value of 0.05 was set as the threshold for significantly different expression by default (*p*-value < 0.05, and *padj* < 0.05). Prediction of the miRNA target gene was performed using psRobot-tar in miRanda for *Sus scrofa*^53^.

### GO and KEGG enrichment analysis of differentially expressed genes

Gene Ontology (GO) functional enrichment analysis and Kyoto Encyclopedia of Genes and Genomes (KEGG) pathway analysis of DEGs were performed using Goseq R package and KOBAS 2.0 software (Available online: http://kobas.cbi.pku.edu.cn/home.do).

### Correlation analysis of DE miRNAs and DE mRNAs

To generate the miRNA–mRNA interaction network, a negative correlation analysis of DE miRNAs and DE mRNAs was performed, and Cytoscape v2.8.3 software (http://www.cytoscape.org/) was used to construct the miRNA–mRNA interaction network.

### Real-time relative quantitative reverse transcription polymerase chain reaction (qRT-PCR)

To validate the sequencing data, 6 DE mRNAs and 6 DE miRNAs were randomly selected and analyzed using real-time relative qRT-PCR and three independent biological replicates were performed at minimum.

For detection of DE mRNAs, RNA was reverse transcribed into single strand cDNA using Prime Script RT reagent Kit RR036A (TaKaRa, Shiga-ken, Japan) on a S1000 Thermal Cycler (Bio-Rad, CA, USA). The GAPDH gene was used as the internal control. The primers used are listed in Supplementary Table S13.

For detection of DE miRNAs, RNA was reverse transcribed into single strand cDNA using PrimeScript RT reagent Kit RR037A (TaKaRa, Shiga-ken, Japan) on a S1000 Thermal Cycler (Bio-Rad, CA, USA). Hieff qPCR SYBR Green Master Mix (Low Rox Plus) (YEASEN, Shanghai, CHN) was used to perform qRT-PCR. The expression of U6 small nuclear RNA (snRNA) was used as the endogenous control. The primers used are listed in Supplementary Table S13.

Real-time relative qRT-PCR was performed in a 10 μL volume containing 100 nM of each of forward primer and reverse primer, cDNA template, and Faststart DNA Master SYBR Green I Mix reagent kit (Roche) on a ViiA 7 Real-Time PCR System (Life Technologies, CA, USA) under the following cycle conditions: 95 °C for 10 min, 40 cycles with 95 °C for 15 s, 56 °C for 30 s, and 72 °C for 31 s.

The relative expression of each target was calculated using the 2^-ΔΔCT^ method with a control group as calibrator^54^. Statistical significance was determined by Student’s *t*-test, with *p*-value < 0.05 deemed to be statistically significant.

## Supplementary Information

Supplementary Information 1.

Supplementary Information 2.

Supplementary Information 3.

Supplementary Information 4.

Supplementary Information 5.

Supplementary Information 6.

Supplementary Information 7.

Supplementary Information 8.

Supplementary Information 9.

Supplementary Information 10.

Supplementary Information 11.

Supplementary Information 12.

Supplementary Information 13.

## Data Availability

The datasets generated and analysed during the current study are available from the corresponding author on reasonable request.

## Code availability

Any custom code used during the current study is available from the corresponding author on reasonable request.
